# A simple PCR-based method to follow and genotype alleles with single nucleotide changes

**DOI:** 10.17912/micropub.biology.000218

**Published:** 2020-02-12

**Authors:** Marie-Charlotte Morin, Sarah Hoff-Yoessle, Sophie Jarriault

**Affiliations:** 1 Université de Strasbourg, 1 rue Laurent Fries, 67404 Illkirch-CU Strasbourg, France; 2 IGBMC, Development and Stem Cells Department, CNRS UMR7104, INSERM U1258

**Figure 1:**
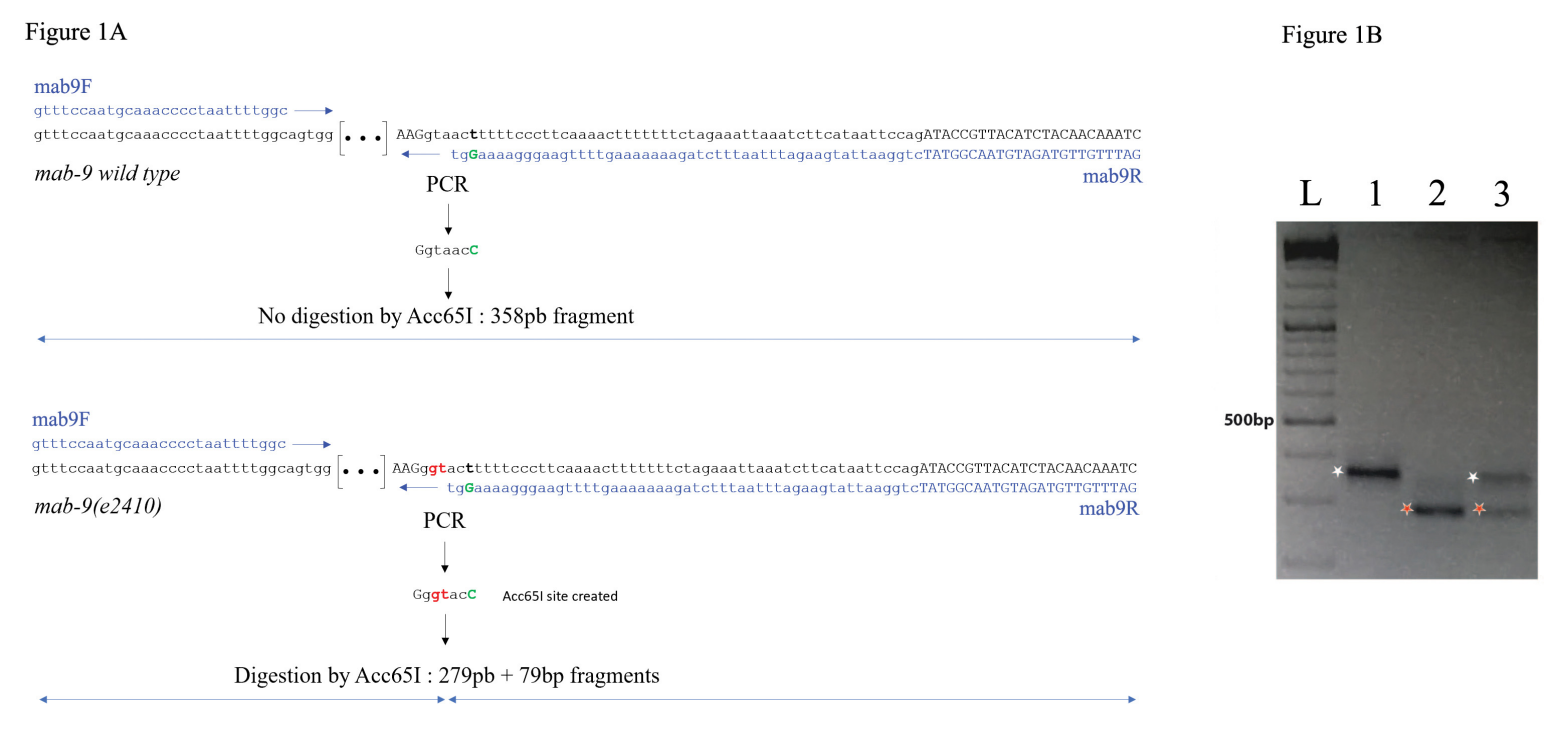
PCR-based detection of homozygous wild type and mutant alleles for *mab-9*. (**A**) primers design allowing the introduction of an Acc65I site in the PCR product from the mutant *e2410* allele. Blue nucleotides: sequence of the primers used; Red nucleotides: DNA alterations in the *e2410* allele; Green nucleotide: PCR-introduced change. Small lettering, intronic sequence; capitals, exons*.* (**B**) Representative analysis on a 2,5% agarose gel. Lane L: ladder, the 500pb band is indicated; lanes 1: WT allele; lanes 2: mutant allele; lane 3: heterozygote animal. The *mab-9* fragment amplified from the WT allele is left undigested by Acc65I with a size of 358pb (white star), while the PCR fragment length obtained from mutant *e2410* allele is 279pb after Acc65I digestion (red star, the remaining 79pb fragment is not visible here).

## Description

Mutant analysis and the building of complex strains bearing several mutant alleles and/or transgenes are precious tools routinely used in studies using genetic models such as *C. elegans*. Crossing, strain building and genotyping necessitate to follow the various alleles to be combined during the crosses, some of which may not exhibit any easily detectable phenotype.Phenotypic markers (usually associated with an obvious phenotype: e.g. *dpy, unc, rol, lon, etc*) have been used as balancers in trans to follow a mutation of interest. This strategy is best used when the marker gene is located close enough to the mutation that one wishes to follow, to avoid crossing overs and recombinations during the several crosses and homozygosing steps. In recent years, using *MosSCI* insertions, Christian Frokjær-Jensen *et al.* have generated a set of strains bearing fluorescent markers inserted at specific chromosomal locations that can also be used as dominant genetic markers (Frokjaer-Jensen **et al.** 2014).

However, depending on the location of the mutation of interest on a chromosome, there might not be any close genetic marker that can be used. In addition, the use of genetic markers to follow a mutation of interest imposes to first build one or several intermediate strains to associate two or more mutant alleles. To introduce a chromosome-integrated transgene in the background of a mutant, strategies using genetic markers can also be limited: the phenotypes of different genetic markers are not all compatible, and the fluorescent expression in MosSCI strains can preclude detection of other transgenes needed in the strain of interest. Finally, other species, from other worms to vertebrates, may not have the same tools such as the extensive *C. elegans* genetic markers and mutant strains library.

Here, we describe a simple and quick method to follow mutations through the several crossing steps. This method is particularly handy when the mutation of interest does not create or modify a restriction site, and avoids having to resort to a multi-step process involving the individual sequencing of multiple animals, after amplification of the genomic region of interest. It is based on the creation of a restriction site by PCR specifically in the amplicon for one of the alleles only, using specific oligonucleotides to amplify a small DNA fragment around the mutation to be followed, and can be applied to several mutations in parallel. This method which uses only two primers for a given gene, one round of PCR and just one PCR product for each allele is also simpler and more robust than the tetra primer ARMS-PCR previously described (Ye **et al.**. 2001).

To date, we have applied this method to the genotyping of 116 mutant alleles, including *daf-2(e1370), ceh-6(gk665), sem-4(n1971, n2654 & n1378), mab-9(e2410), eri-1(mg366), egl-5(n945), egr-1(ku285), jmjd-3.1(fp15), wdr-5.1(ok1417)*. The results for *mab-9(e2410)* are shown in figure 1 and have been repeated 5 times.

*mab-9* is T-box transcription factor well studied for its role in hindgut and tail development. Unfortunately, this gene is in a genomic region poor in phenotypic balancer genes, and shows a subtle mutant phenotype under dissecting microscope (https://wormbase.org/species/c_elegans/gene/WBGene00003106#0-9f-3). The *mab-9(e2410)* allele exhibits two point mutations (TA to GT) in the 4^th^ intron, that cause a splice defect (Woolhard & Hodgkin 2000, Figure 1A). The WT sequence is gtaact and the *mab-9(e2410)* sequence g**GT**act. To follow this mutant allele during crosses, it is possible to modify one nucleotide by PCR and specifically create an Acc65I site (G/GTACC) in the amplicon from the mutant allele: By replacing in the mab9R reverse primer the last T of the mutant g**GT**act sequence by a C, an Acc65I g**GT**ac**c** site is created in the mutant amplicon only (Figure 1A). Primers mab9F and mab9R allow amplification of a 358 bp PCR fragment. After Acc65I digestion, the PCR product from *mab-9(e2410)* is digested into a 279bp and a 79bp fragments, while the WT version remains 358pb, and heterozygous animals exhibit three bands at 358bp, 279bp, and 79bp (Figure 1B). Using this method, genotyping is achieved in a couple of hours enabling to pick the desired animals the same day. Since it bypasses the need to build genetically balanced strains, the time to obtain the final strain is considerably reduced. This PCR-based strategy is widely adaptable to many other organisms than *C. elegans*, from well-known genetic models such as drosophila, zebrafish or other worms to models lacking a full genetic tool box.

## Methods

This method typically allows the amplification of a short fragment (around 350 pb +/-50bp) with the mutation of interest. Here is a brief description of the workflow:

**1. Design oligonucleotides**: locate the SNP between the wild type and the mutant alleles on the locus sequence. Design a pair of amplifying primers with one of them ending its 3’ extremity just before the SNP. By changing a single nucleotide (or two) close to the mutation of interest directly on this primer, it is possible to create a restriction site. For example, in the sequence nnGTTAA**A**nn (were the last A would be the point mutation in the mutant allele to follow), it is possible to create a palindromic sequence in changing the G by a T. Like most of the 6 nucleotides palindromic sequences, TTTAAA correspond to a restriction site: the DraI site here. Thus, if the WT has the sequence nnGTTAAGnn and the mutant nnGTTAA**A**nn, after amplification and DraI digestion, the following sequences will be obtained: nnTTTAAGnn for the WT amplicon (uncut) and nn**T**TTAA**A**nn the mutant one (cut). Designing which restriction enzyme site to introduced can be facilitated by using the WatCut versatile resource for the use of restriction enzymes in molecular biology experiments (http://watcut.uwaterloo.ca/template.php?act=snp_new). If you are using the SNP-RFLP WatCut help, please make sure you try different parameters: number of base change (starting with 1 is recommended), as well as distance to SNP(s). Please make sure the primer used to introduce or change a restriction site extends by at least 2 nucleotides 3’ of the introduced sequence change, and make sure the amplified fragment is around 350pb (+/-50bp). Since the size difference after digestion will be the length of the primer which 3’ extremity is close to the variation, we recommend to design a longer (50nt or so) primer. We would not recommend to go below 30 nucleotides in length for convenient detection on agarose gels. Finally, to ensure that you are following your mutant allele, it might be more adequate to introduce the restriction site using the mutant sequence.

**2. Prepare worms lysates**; we have used this method to follow mutations during crosses, and thus have routinely used single worm lysates (http://www.wormbook.org/chapters/www_SNPsintrotwopointmap/SNPsintrotwopointmap.html#d0e185)) for our PCRs, picking adults after they have laid eggs for 2-3 days. It is recommended to run PCRs on WT and mutant samples in parallel, as well as on a mix of a mutant and a wild type worm to visualise heterozygote animals.

**3. Run the PCRs**

**4. Digest the PCRs** using the restriction enzyme which site is created when amplifying one of the alleles.

**Run a 2% to 3% agarose gel** (the exact concentration will depend on the size of the two fragments to detect: full length PCR, versus full length PCR minus the length of the oligonucleotide including the restriction site).
